# Adipose Tissue Dynamics: Cellular and Lipid Turnover in Health and Disease

**DOI:** 10.3390/nu15183968

**Published:** 2023-09-14

**Authors:** Ivonne Palacios-Marin, Dolors Serra, Josep Jimenez-Chillarón, Laura Herrero, Marijana Todorčević

**Affiliations:** 1 Endocrinology Department, Institut de Recerca Sant Joan de Déu, Esplugues, E-08950 Barcelona, Spain; 2 Department of Biochemistry and Physiology, School of Pharmacy and Food Sciences, Institut de Biomedicina de la Universitat de Barcelona (IBUB), Universitat de Barcelona, E-08028 Barcelona, Spain; 3 Centro de Investigación Biomédica en Red de Fisiopatología de la Obesidad y la Nutrición (CIBEROBN), Instituto de Salud Carlos III, E-28029 Madrid, Spain; 4 Department of Physiological Sciences, School of Medicine, University of Barcelona, E-08907 L’Hospitalet, Spain

**Keywords:** adipose tissue, obesity, lipid turnover, adipocytes turnover

## Abstract

The alarming increase in obesity and its related metabolic health complications, such as type 2 diabetes, has evolved into a global pandemic. Obesity is mainly characterized by excessive accumulation of adipose tissue, primarily due to an imbalance between energy intake and expenditure. Prolonged positive energy balance leads to the expansion of existing adipocytes (hypertrophy) and/or an increase in preadipocyte and adipocyte number (hyperplasia) to accommodate excess energy intake. However, obesity is not solely defined by increases in adipocyte size and number. The turnover of adipose tissue cells also plays a crucial role in the development and progression of obesity. Cell turnover encompasses the processes of cell proliferation, differentiation, and apoptosis, which collectively regulate the overall cell population within adipose tissue. Lipid turnover represents another critical factor that influences how adipose tissue stores and releases energy. Our understanding of adipose tissue lipid turnover in humans remains limited due to the slow rate of turnover and methodological constraints. Nonetheless, disturbances in lipid metabolism are strongly associated with altered adipose tissue lipid turnover. In obesity, there is a decreased rate of triglyceride removal (lipolysis followed by oxidation), leading to the accumulation of triglycerides over time. This review provides a comprehensive summary of findings from both *in vitro* and *in vivo* methods used to study the turnover of adipose cells and lipids in metabolic health and disease. Understanding the mechanisms underlying cellular and lipid turnover in obesity is essential for developing strategies to mitigate the adverse effects of excess adiposity.

## 1. Introduction 

White adipose tissue (WAT) was previously considered to be a simple, passive, lipid storage tissue for many years. With obesity rates rapidly increasing over the last several decades, WAT is now recognized as a highly complex metabolic and endocrine organ involved in energy regulation, as well as cardiovascular and immune function, and reproduction. 

A continuous oversupply of food leads to the expansion and remodelling of adipose tissue (AT), weight gain, and obesity, ultimately leading to several metabolic diseases, such as type 2 diabetes, cardiovascular disease, hepatic steatosis, and some types of cancer [1,2,3,4]. Increased fat accumulation in later life is also linked to changes in brain structure and function, commonly observed alongside cognitive decline, dementia, and Alzheimer’s disease (AD) [5].

The alarming increase in obesity prevalence has fuelled studies into WAT growth and dynamics in recent years. To fully understand WAT growth, it is necessary to study both the lipid and cellular dynamics within each depot at different life stages. Although a large number of studies have characterized WAT metabolism and signalling *in vitro*, very few have assessed WAT dynamics *in vivo*. A better understanding of adipose dynamics in health and disease may lead to novel strategies to prevent and treat obesity. Here, we aim to comprehensively review the knowledge on WAT and lipid turnover in health and disease. 

## 2. AT Heterogeneity

### 2.1. From Brown to Pink Adipocytes

In mammals, AT is normally classified according to its localization and morphophysiological properties into white (WAT), brown (BAT), beige, and pink AT. WAT is the most abundant type of AT found in adult humans and is the main tissue involved in energy storage. White adipocytes are characterized by a large unilocular lipid droplet that occupies 95% of the cell volume, consequently determining cell size [1], and a low number of mitochondria. Contrary to white adipocytes, brown adipocytes contain multilocular lipid droplets, are rich in mitochondria, and highly vascularized. Brown adipocytes express uncoupling protein 1 (UCP1), which is responsible for uncoupling oxidative phosphorylation to produce heat to maintain body temperature [2]. BAT was previously only considered to be important in rodents and newborn humans; however, around a decade ago, several studies showed that adult humans also possess distinct metabolically active BAT depots [2]. Beige adipocytes, yet another type of adipocyte, are believed to be formed by a phenomenon referred to as “browning”, which is defined as an increase in UCP1 mRNA expression. It has been suggested that beige adipocytes predominantly arise in WAT depots in response to various activators, such as cold or β-adrenergic stimulation [6]. Both brown and beige adipocytes have gained increased interest as potential targets for the treatment of obesity and associated metabolic disorders. Finally, pink adipocytes are formed in females during pregnancy, lactation, and post lactation. Subcutaneous white adipocytes are converted to pink adipocytes, which then serve as milk-producing glands formed by lipid-rich elements [3]. 

Taken together, these findings show that adipocytes possess a high capacity for cellular plasticity and can undergo striking physiological changes in response to environmental stimuli, where white adipocytes can transdifferentiate to form brown, beige, or pink adipocytes under specific conditions [3,4,7,8,9]. 

### 2.2. WAT: Different Locations, Different Rules

**Figure 1:**
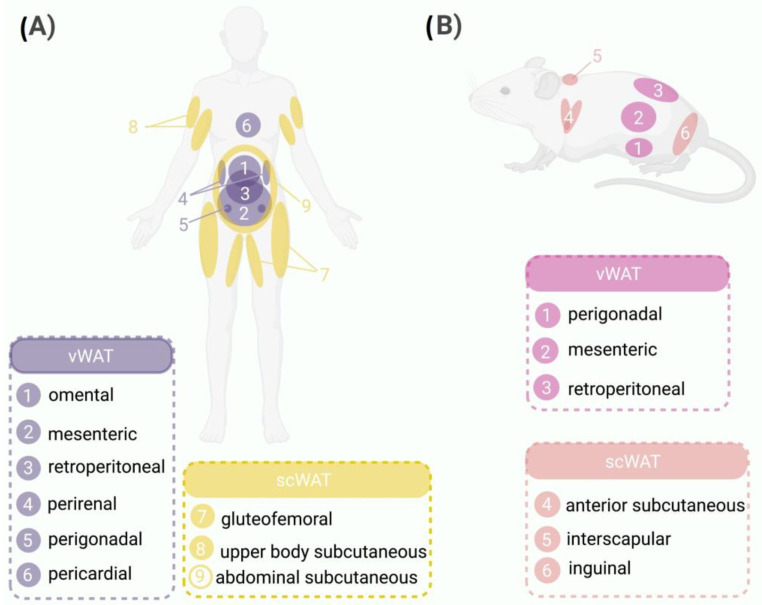
WAT distribution in humans and mice. Comparison between the distribution patterns of WAT in humans and mice highlighting the anatomical similarities and differences between the two species. (**A**) Schematic representation of WAT distribution in humans highlighting the major depots, such as vWAT surrounding internal organs (1, omental; 2, mesenteric; 3, retroperitoneal; 4, perirenal; 5, perigonadal; 6, pericardial) and scWAT located beneath the skin (7, gluteofemoral; 8, upper body subcutaneous; 9, abdominal subcutaneous). (**B**) WAT distribution in mice, showing vWAT located in the abdominal region (1, perigonadal; 2, mesenteric; 3, retroperitoneal) and scWAT situated under the skin (4, anterior subcutaneous; 5, interscapular; 6, inguinal).

In humans, WAT is classified according to body localization into two categories: (1) visceral WAT (vWAT), which is located intra-abdominally and includes omental, mesenteric, retroperitoneal, perirenal, gonadal, and pericardial WAT; and (2) subcutaneous WAT (scWAT) [10], which is subdivided into gluteofemoral subcutaneous adipose tissue (gSAT; lower body regions in the thighs, hips, and buttocks), upper body subcutaneous adipose tissue (arms, trunk, and abdomen), and abdominal subcutaneous adipose tissue (aSAT) (Figure 1A).

In rodents, WAT is functionally distinct to that in humans and is divided into five different depots: (1) perigonadal WAT, which is known as periovaric WAT in females (surrounds the uterus and ovaries) and epidydimal WAT in males (surrounds the epididymis and testis); (2) retropetironeal WAT, which is located within the abdominal cavity along the dorsal wall of the abdomen behind the kidneys; (3) mesenteric WAT, which lines the surface of the intestines; (4) scWAT, which is classified as anterior subcutaneous WAT (located in the interscapular and axillary areas) and interscapular WAT (located between the scapulae); and (5) inguinal WAT, which is attached dorsally along the pelvis to the thigh of the hindlimb [11]. 

The metabolic, physiological, and molecular characteristics of WAT vary between depots and even within them [10]. The exact mechanisms that drive these differences are not fully understood. Several mechanisms explaining the differences between depots have been proposed, ranging from the anatomical localizations of vWAT and aSAT, which favour the secretion of adipokines and free fatty acids to the portal blood stream compared with gSAT, to a series of intrinsic biological differences in adipocytes regarding hormone and cytokine production, cellular receptors, and responsiveness to other remotely produced hormones [12]. 

Several in-depth studies have reported that differences in the distribution of adipocyte size and average area between visceral and subcutaneous depots influence adipocyte function [13]. For example, the synthesis and secretion of adipokines differ between the two depots [12,14]. Leptin, an adipokine that plays a key role in energy homeostasis as well as endocrine and immune functions, is produced and secreted in subcutaneous adipocytes at higher rates than visceral adipocytes [15]. On the other hand, adiponectin, an adipokine that is closely related to degree of hyperinsulinemia and insulin sensitivity [16,17], is produced in higher quantities in vWAT than scWAT [14]. Therefore, the production of these metabolic molecules is often altered in obesity, a state that has long been associated with the presence of hypertrophic adipocytes [18]. 

One of the key unsolved questions is what exact mechanisms determine these differences between AT depots. It has been proposed that intrinsic differences within adipocytes may rely on their ontogeny [11,19]. Recent findings further support the existence of different subgroups within white adipocytes, found in both human and murine WAT. These specific subtypes of adipocytes have potential to possess unique functional traits, contributing to multifaceted roles in the functionality of WAT and the onset of metabolic disorders linked to obesity [20]. 

In addition, new research has indicated that the different dynamics of AT growth, as well as fluctuations in adipose cell size and lipid turnover, may contribute to distinct responses of different AT depots in health and disease.

## 3. Dynamics of AT 

WAT expansion during development is accomplished through increases in both adipocyte number (hyperplasia) and cell size (hypertrophy) [13,21,22,23,24,25,26]. Differences between adipocytes and lipid turnover rates in WAT may contribute to depot-specific fat cell size and number differences, potentially contributing to metabolic complications in individuals who are overweight and obese. Fat mass is determined by the net balance between the storage of new triacyclglycerol (TAG; lipogenesis) and removal of previously stored TAG (lipolysis) in combination with the recruitment of new functional adipocytes (adipogenesis) [21,22,24,25,27]. 

### 3.1. Lipid Turnover

Lipid turnover is an important factor to study in order to understand AT dynamics. Using gas–liquid chromatography, it was established that AT fatty acid composition reflected dietary fatty acid composition [28]. The time required for the fatty acids in WAT to change in response to dietary composition was shown to be around 6 months [28,29], and de novo lipogenesis (DNL) only contributed to a minor part of stored fat [28]. In recent years, great efforts have been made to find a non-invasive way to trace the lipid components of WAT [30]. The slow lipid turnover described in human WAT [28] makes it difficult to find a suitable non-harmful isotope that can be maintained in WAT for several months. Nevertheless, the use of deuterium (^2^H) present in ^2^H_2_O has recently helped to address this [29,30,31,32,33]. Due to its non-toxic nature, ^2^H is safe and easy to administer through drinking water, inexpensive, and suitable for long-term studies [29,30].

Strawford et al. [29] measured the incorporation of ^2^H from ^2^H_2_O into the glycerol in TAG (representing TAG turnover), palmitate in TAG (representing DNL), and cellular DNA (representing cell proliferation) using gas chromatography/mass spectrometry (GC/MS) in lean humans after exposure for 5 and 9 weeks. The authors reported that whole-body ^2^H_2_O enrichment levels were very stable. The mean TAG glycerol synthesis rate was 12%, net lipolysis was 50–60 g/day, and the half-life of TAG was 6–9 months. Label decay measurements at 5–8 months after discontinuing ^2^H_2_O showed similar turnover estimates [29]. The authors reported that DNL contributed 20% of newly deposited TAG in WAT, which was slightly higher than previously reported [28,29]. In conclusion, overall scWAT lipid turnover rates in normal-weight men and women were slow and stored lipids had a long half-life [28]. In lean individuals, fat is primarily stored in subcutaneous depots, which have a lower rate of fat synthesis (lipogenesis) and breakdown (lipolysis) compared with visceral depots. Therefore, when estimating lipid turnover in lean individuals, the exclusion of visceral AT depots is unlikely to have a significant impact. However, it is important to note that these findings cannot be generalized to populations with obesity or abdominal obesity. Considering the amount of data showing that vWAT and scWAT behave differently [33,34,35,36,37,38,39], we argue that further studies into lipid turnover in both types of depots are essential, especially in an obesity scenario. 

Most previous studies into *in vivo* WAT dynamics have been limited by short-term experimental conditions [29,33,40,41,42,43], providing a limited amount of information. To address this problem, Spalding et al. developed an interesting method that retrospectively determined the age of adipocytes and their lipid components (TAG) through radiocarbon dating using atmospheric ^14^C released during nuclear testing in the Cold War [44,45]. The authors reported a positive correlation between fat cell volume and total body fat mass in adult humans, thus indicating that the lipid content of adipocytes has a strong impact on total body fat. Since the relationship between cell size and total body fat was not linear, the authors concluded that in addition to cellular lipids, adipocyte number was a major determinant of fat mass during adulthood [45]. Using the same method, Arner et al. [44] dated TAG from scWAT adipocytes in a larger cohort that included both lean and participants with obesity as well as those with familial combined hyperlipidaemia (FCHL). The results showed that mean lipid age, defined as the average time that lipids spent in WAT, of stored TAG was 1.6 years in lean participants [44]. Interestingly, lipid age was not related to adipocyte size and showed the least variability between different fat depots and within individuals regardless of their weight [44,45]. Thus, there is a continuous exchange of lipids between adjacent adipocytes in WAT, and this exchange is not dependent on adipocyte size [44]. Furthermore, the turnover of lipids in WAT remains highly consistent throughout adulthood and shows no variation based on age or sex [44].

More recently, the first long-term longitudinal study that measured lipid turnover of human WAT was reported [46]. By assessing the ^14^C/^12^C ratio in the lipids of abdominal scWAT, the mean lipid age, lipid removal rate, and net lipid uptake were determined in normal-weight adult women and men. Biopsies of abdominal scWAT were collected from participants who were divided into three categories based on the evolution of their body weight during the follow-up period: weight-stable, weight-gainers, and weight-losers. Strikingly, the authors reported that over the 13-year follow-up period, the lipid age increased significantly by 0.6 ± 0.8 years in most participants, independent of starting age and weight change of the participant, suggesting that lipid removal rate decreases with aging, in contrast to previous cross-sectional reports [44]. Furthermore, participants who displayed no changes in adipocyte lipid uptake during the follow-up period showed an increase in body weight of approximately 20% [46]. Surprisingly, changes in body weight and lipid age were not correlated, and mean lipid age did not differ between “weight-gainers” and “weight-losers”. Nevertheless, when adipocyte lipid uptake was compared among these two groups, “weight-losers” showed a decrease in lipid uptake, suggesting that adipocyte lipid uptake is the most important mechanism through which WAT lipid accretion takes place during adulthood and aging [46]. These recent findings add a new perspective on WAT lipid remodelling and maintenance of body weight during the life course. The aging process appears to slow down WAT lipid turnover and, unless compensated by a reduction in energy intake, results in accretion of fat mass. These recent findings highlight the importance of investigating lipid dynamics in WAT during childhood and adolescence.

### 3.2. Adipocyte Turnover

Under physiological conditions, mature cells either perform their function or activate a death program that involves their replacement in the tissue (turnover) by new cells derived from the stromal vascular fraction (SVF) [3]. This emphasizes that, alongside adipose tissue expansion, adipocyte death holds a crucial role in the turnover of adipose tissue.

Assessment of *in vivo* human cellular turnover in terminally differentiated postmitotic cells, such as adipocytes, faces several methodological limitations. One of the main challenges is the toxicity of radiolabelled nucleotides, which, in combination with the slow turnover rate of WAT components [29,30,47] makes it extremely difficult to label and trace adipocyte and adipose progenitor cell (APC) turnover. As a result, there is a scarcity of information regarding the turnover of these cell types. Strawford et al. [29] used a 9-week ^2^H_2_O labelling experiment to measure the kinetics of the SVF and adipocyte fraction in healthy-weight adults. After determining the cellular kinetics from both fractions in different WAT depots, cellular proliferation was found to be between 10% and 17%, with a mean half-life of 8–14 months. Interestingly, the authors reported that the fractional replacement of cells in the SVF was consistently significantly higher than in the mature adipocyte-enriched fraction, suggesting that most proliferating APCs do not permanently join the mature adipocyte population [29]. Since neither the isolated adipocyte fraction nor SVF were purified during this study, this could have led to overestimation of adipose cellular turnover due to the presence of other non-adipogenic cellular populations (i.e., endothelial or immune cells). This could be especially important in the case of the SVF, which is very rich in non-adipose cell types.

Spalding et al. measured ^14^C-labelled genomic DNA in adipocytes from scWAT to assess adipocyte kinetics in a large cross-sectional cohort of individuals aged between 20 and 70 years [45]. To estimate adipocyte turnover during the life course, the authors combined their data with previous reports of cellularity from children and adolescents [23]. Interestingly, after grouping participants in 5-year bins, adipocyte number levelled off and remained constant during adulthood irrespective of weight category, and no differences in cell death were found across all body mass index (BMI) categories [45]. Based on these findings, it was concluded that differences in adipocyte number among different BMI categories are established earlier in life during certain growth periods, placing childhood and adolescence in the spotlight for the development of WAT. Next, the authors studied whether adipocytes were replaced during adulthood. Surprisingly, ^14^C-labelled genomic DNA levels in all the analysed individuals provided the first indication that there is continuous and substantial turnover of adipocytes in adult humans. Furthermore, 10% of fat cells were replaced annually in all adult ages across all studied BMI categories, and neither cell birth nor death were altered by aging [45]. Based on their developed cell-birth and cell-death model, the authors concluded that the mean age of human adipocytes was 9.5 years, implying that TAG is replaced an average of six times during the lifetime of the adipocyte [44,45]. No correlation between participant age and adipocyte age or cell death rate was found, suggesting that cellular turnover rate remains constant throughout adult life [45].

Overall, available data support the idea that WAT cellular turnover is slow [23,24,36,37,38]. The fact that this conclusion was reached using different methodological approaches makes this hypothesis very strong. Nevertheless, discrepancies regarding scWAT cellular kinetics have been reported [29,30,42,44,45]. This could be attributed to several factors, such as differences in the periods of labelling and tracing, methodologies, study sample size, as well as possible artifacts that may be generated with each methodology. 

## 4. Dynamics of AT in Obesity

Disorders of AT mass, such as obesity, have an underlying kinetic basis. Synthesis and/or breakdown rates of lipids are altered during WAT mass changes. Differentiated adipocytes have remarkable hypertrophic potential, which is very important in states of being overweight and in obesity, in which the intracellular lipid content increases to accommodate the excessive nutrient influx. However, when the maximum storage capacity of adipocytes is reached, lipids then accumulate in the liver, muscle, or heart in a phenomenon referred as ectopic fat accumulation, which leads to lipotoxicity. This has been associated with several metabolic disturbances and postulated as drivers of the development of insulin resistance, metabolic syndrome, and type 2 diabetes [27,37,48,49,50].

Studies performed in humans and rodents in the 1970s established that individuals with obesity had larger adipocytes than lean individuals [18,21,51]. Furthermore, cell size was shown to be variable among individuals within the same group, between different fat depots, and even within depots [18], revealing enormous heterogeneity within adipocyte sizes. Further research supported the idea that in obesity, AT develops a hypertrophic phenotype, which is often accompanied by an increased adipose cell number [23,52]. It remains unclear what signals determine the type of WAT expansion. It has been hypothesized that hyperplastic WAT growth takes place when obesity onset occurs at an early age, whereas hypertrophic growth takes place at all life stages and may be the main mechanism for WAT growth when obesity onset occurs at a later age [23,26,30,33,45,52,53].

### 4.1. Lipid Turnover in Obesity

Recent cross-sectional studies have shown that obesity is associated with increased adipocyte lipid deposition and low adipocyte lipid removal rate, which reflects a higher lipid age of WAT lipids in both overweight and obese individuals [39,44,54]. Together, these disturbances promote the development and maintenance of excessive fat mass. The mean lipid age of scWAT in individuals with obesity was ~0.6 years higher than in individuals with a BMI ≤25 kg/m^2^ [39], suggesting that lipid turnover is slower in overweight and obesity conditions. The authors did not find any significant differences between male and female participants regarding WAT lipid dynamics [39]. 

Lipolysis is the first step in the removal of adipocyte lipid and the ability to stimulate lipolysis has been measured *in vitro* in human isolated adipocytes and compared with *in vivo* measurements of lipid turnover [44,54]. The *in vitro* stimulated rate of lipolysis was found to be positively correlated with adipocyte TAG removal rate and inversely correlated with lipid age in normal-weight and individuals with obesity [44], thus suggesting that impaired lipolysis may be an important mechanism for excessive fat mass accrual. The ability of different lipolytic agents to stimulate lipolysis and glycerol release *in vitro* from abdominal subcutaneous adipocytes isolated from normal-weight and individuals who are overweight showed that induced lipolysis was inversely correlated with TAG age and BMI [54]. Furthermore, adipocyte lipid turnover and lipolytic activity were decreased in overweight individuals and individuals with obesity and reflected current BMI [54], suggesting that disturbances in scWAT lipid dynamics take place very early in the development of excessive fat mass [39,44,54]. These results obtained in adult humans add a new perspective to the obesity WAT lipid dynamics, where in addition to an increased adipocyte lipid uptake, impaired lipolysis and a decreased ability to export lipids contributes to the development or maintenance of excessive fat mass [39,54].

In contrast to the disturbances observed in scWAT lipid dynamics, it has been reported that the TAG age of vWAT depots did not change in overweight or obesity conditions, and normal TAG turnover was maintained in vWAT depots across a broad range of body fat levels [39]. vWAT lipid age was only altered in severe obesity, in which the TAG age was ~0.7 years higher, suggesting that vWAT lipid turnover is only reduced in severe obesity [39]. Furthermore, individuals with central or pronounced visceral obesity showed a higher lipid uptake and ~0.7-year younger TAG age in vWAT depots, suggesting that TAG turnover in vWAT depots is increased in abdominal or central obesity [39]. In summary, the current evidence suggests that visceral and subcutaneous WAT depots possess a distinct *in vivo* lipid turnover. 

#### Lipid Turnover and Metabolic Health

Adipocyte morphology has been linked not only to excessive fat mass, but also metabolic disorders and dysfunction in AT [34,35,55,56,57,58]. A “worse” adipocyte morphology (hypertrophy), which is characteristic of obesity, has been linked with fasting plasma insulin levels and insulin sensitivity [27,42,50,59]. Hypertrophic adipocytes display disturbances in cell metabolism, increased basal lipolysis, and increased leakage of free fatty acids [60], whereas smaller insulin-sensitive adipocytes show a higher lipogenesis-to-lipolysis ratio [61]. Since lipid turnover determines adipocyte size, lipid dynamics should be tightly related in line with systemic metabolic homeostasis. Several correlations between biomarkers and scWAT lipid kinetics have been reported, such as fractional TAG synthesis with waist-to-hip ratio and DNL with plasma glucose concentration [29]. 

**Figure 2:**
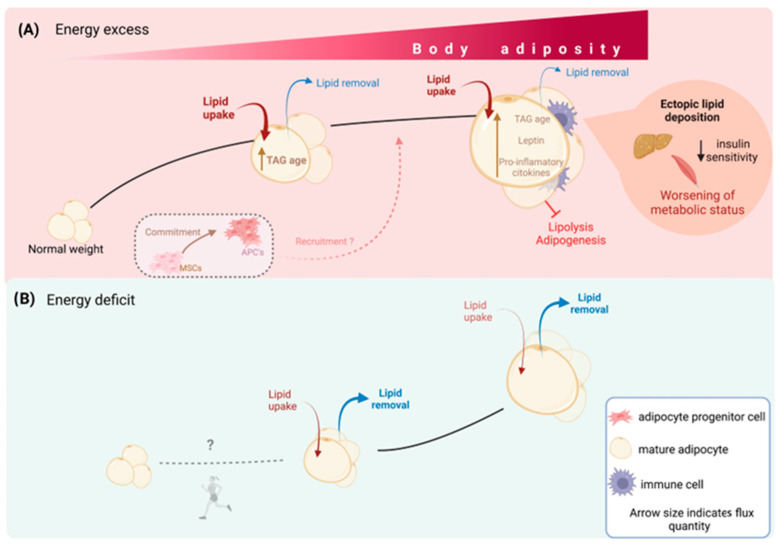
WAT dynamics determine metabolic health. Lipid and cellular turnover in WAT determine the organ size. In situations of excess energy availability (**A**), adipocytes actively take up lipids and store the surplus energy as TAG, while the removal of lipids from adipocytes is minimal. Consequently, the average age of TAG within the adipocytes increases, leading to an overall increase in body weight and fat mass. Recruitment of adipocyte progenitor cells to generate new adipocytes is believed to meet the energy storage demands primarily in childhood, but it is unclear whether this mechanism persists during adulthood. On the other hand, in situations of energy deficit (**B**), lipid uptake by adipocytes decreases, while the removal of lipids increases. As a result, adipocyte size decreases and total body fat mass also decreases. No evidence suggests that energy restriction decreases the number of adipocytes, which are believed to remain constant after growth periods. Exercise may reduce new adipocyte formation, especially in animal models, but it does not decrease the total number of established adipocytes.

Based on the expandability hypothesis of AT [50], it was investigated whether an attenuated ability of scWAT to store TAG is associated with a pathophysiological influence. Subcutaneous fat cell size and lipid age were compared in individuals with “healthy” or “unhealthy” obesity according to the ATPIII criteria [39]. Despite similar BMI and abdominal scWAT mass between groups, individuals with “unhealthy” obesity displayed a worse adipocyte morphology and had fewer but larger fat cells than individuals with “healthy” obesity. Nevertheless, no interaction between adipocyte size and lipid age was found in any of the groups [39]. However, when participants were categorized according to subcutaneous adipocyte cell size, a significantly increased TAG age was reported in individuals with “unhealthy” obesity who had a relatively small adipocyte size, suggesting that low adipocyte lipid removal is present in “unhealthy” obesity independent of adipocyte cell size [39]. These findings suggest that differences between individuals with “healthy” and “unhealthy” obesity cannot merely be explained on the basis of fat cell size and instead adds another factor to the relationship between WAT dynamics and metabolic health, in which a decrease in lipid turnover is a major driver of metabolic disturbances [39]. In this scenario, ectopic fat deposition and metabolic disturbances result from a combination of an insufficient ability of scWAT to store TAG and an attenuated adipocyte lipid removal rate (Figure 2A). The fact that scWAT seems to reach a maximal capacity to store and release lipids in the transition from normal weight to overweight [54] may explain why lipids accumulate in the liver even when only a moderate excessive adiposity has developed [39]. Other studies have concluded that adipocyte size is heterogeneous and displays bimodal distribution, in which larger adipocytes from aSAT and decreased expression of differentiation markers have been positively associated with metabolic dysfunction [13,62,63]. Although discrepancies exist between studies [13,29,39], altered scWAT lipid turnover is a major driver for obesity-related metabolic disturbances. 

To further investigate the relationship between WAT lipid kinetics and metabolic homeostasis in the absence of obesity, the lipid turnover of scWAT from normal-weight individuals was compared with that in normal-weight individuals with familial combined hyperlipidaemia (FCHL) [44]. The lipid age of stored TAG was markedly increased in the individuals with FCHL compared with those without FCHL. Although the participants with FCHL were not obese, the lipid age of this group was very similar to that of participants with obesity without FCHL [44]. Adipocyte lipid uptake in participants with FCHL was decreased compared with lean individuals that did not present FCHL. The combination of low lipid uptake and low lipid removal rate resulted in a reduced lipid turnover of WAT in individuals with FCHL. Low WAT lipid turnover may promote the shunting of fatty acids to the liver [64], where they will drive the development of dyslipidaemia and other metabolic disturbances [44,64]. A correlation between TAG age and insulin resistance, assessed using homeostatic model assessment for insulin resistance, was reported in individuals with obesity but without FCHL and lean individuals with FCHL, indicating that the lipid removal rate in WAT has an impact on body insulin sensitivity independent of underlying disorder [44]. These findings show that lipid turnover is another independent regulator of overall metabolic homeostasis. 

### 4.2. Adipocyte Turnover in Obesity

Due to their terminally differentiated nature, it was long thought that human adipocytes could not be acquired during adulthood and WAT expansion occurred via hypertrophic growth (i.e., obesity) [51]. Nevertheless, several studies have reported that increased adipocyte volume does not entirely account for the variability in fat mass either within or between lean individuals or those with obesity [26,45]. The identification of APCs in adult human AT [65] provided a mechanism by which adipocytes could be acquired at any stage of life and raised further questions about the mechanisms involved in WAT dynamics within this period of life. 

Spalding et al. assessed the *in vivo* cellular kinetics of scWAT in lean adult individuals and adult individuals with obesity [45]. They reported no differences in adipocyte death rate in scWAT from individuals with obesity when compared with lean controls. Nevertheless, individuals with obesity were found to have a significantly greater number of adipocytes added per year in scWAT. Strikingly, no increase in average cell number in individuals with obesity was found and the proportion of newly born adipocytes was similar between both groups. It was argued that the adipocyte birth rate must match the death rate to maintain the proportion of newborn adipocytes added each year (turnover) for all weight categories [45]. Taken together, these findings led the authors to conclude that differences in cellularity between individuals with or without obesity occurred before adulthood.

By integrating data from individuals of all ages, it was determined that an increase in adipocyte number occurred significantly earlier in individuals with obesity. Furthermore, the acquisition of adipocytes was higher (augmented relative acquisition) and the end of the expansion of adipocyte number occurred earlier in obesity [45]. Thus, it was concluded that, in adults, the adipocyte number was set earlier in individuals with obesity and was not caused by a prolonged expansion period. Overall, the number of adipocytes is subject to little variation during adult life; thus, although there is substantial adipocyte turnover during adulthood, the total cellularity is tightly controlled within this period and no important variations occur despite remarkable changes in adipocyte size and total fat mass. Although evidence pointed towards earlier life as the period that most likely contributes to increased cellularity, based on the cross-sectional design of the study, it could not be excluded that individuals who gradually gain significant weight over the years may initially increase their adipocyte size until a threshold is reached [45], after which recruitment of new fat cells from committed precursor cells or mesenchymal stem cells could take place. 

Using a different approach, White et al. assessed the *in vivo* cellular kinetics of aSAT and gSAT in women who are overweight and obese [33]. They performed a short-term (8 weeks) ^2^H cell-labelling experiment using heavy water (^2^H_2_O) intake. The authors determined the cellular kinetics using GC/MS and a linear mixed model of APC proliferation and adipocyte fractions of both depots. The DNA synthesis rate denoted the formation of adipocyte and adipocyte progenitors in aSAT and gSAT, and adipocyte formation in both depots was positively correlated with APC formation and related to body fat percentage [33]. In contrast to previous reports using animal models [66], the authors reported that the formation of adipocytes and APCs was higher in gSAT compared with aSAT. Interestingly, as previously reported [29], preadipocyte formation was greater than adipocyte formation [33]. Likewise, the new preadipocyte-to-adipocyte ratios were correlated between depots. This may suggest constant preadipocyte recruitment to accommodate energy surplus, and since adipocytes arise from preadipocytes, one would expect an increase in APCs to precede adipocyte formation [33]. In line with previous findings [45], no significant impact of BMI on overall WAT kinetics was found. Interestingly, despite no correlation between BMI and WAT cell proliferation, preadipocyte and adipocyte proliferation in aSAT correlated with total body fat percentage [33]. Overall, using this ^2^H DNA-labelling approach, the authors showed that APC proliferation in aSAT and gSAT depots in women who are overweight and obese was correlated with the formation of adipocytes, although not all the newly recruited APCs joined the mature adipocyte fraction. Despite the lack of differences in cellular kinetics reported across BMI categories, preadipocyte and adipocyte kinetics were highly correlated with body fat content [33].

Using the AdipoChaser mouse model, Wang et al. studied the effects of acute and chronic hypercaloric intake on de novo adipogenesis in the subcutaneous and visceral WAT of adult mice. By chasing newly formed adipocytes, the authors showed that when challenged with a high-fat diet (HFD) for a short period, the adipocytes from both depots increased markedly in size, reflecting a high capacity for adipocyte hypertrophy to cope with acute hypercaloric intake. Interestingly, when the HFD stimulus was maintained for longer, vWAT showed a higher rate of adipogenesis than scWAT [66]. These findings showed that, in rodents, excessive nutrient intake during adulthood results in WAT expansion initially through hypertrophy; however, if the positive energy balance becomes chronic, vWAT and scWAT cope with it differently, uncovering a differential *in vivo* adipogenic capacity among WAT depots. In agreement with this hypothesis, others have reported similar findings in high-fat fed C57Bl/6 adult mice, which exhibited increased adipogenesis in vWAT after long-term exposure to HFD, suggesting that mice exhibit selective remodelling of WAT depots in response to chronic nutritional imbalance [53]. However, the point at which AT loses its ability to expand is not clear. There is evidence to suggest that a combination of marked adipocyte hypertrophy, impaired lipolysis, and decreased adipogenesis rate are all important factors in total body fat mass and turnover dynamics [39,44,45,46,52,54]. The use of animal models in this important field is proving to be very helpful to better understand the mechanisms involved.

#### Adipocyte Turnover in Unhealthy Metabolic State

It has been proposed that hypertrophic adipocytes secrete paracrine factors that promote APC recruitment and adipocyte differentiation [27,59,67], suggesting hyperplasia as a “recovery mechanism” to ameliorate overnutrition-induced metabolic disturbances [45,66]. Hypertrophic “unhealthy” adipocytes show a proinflammatory adipokine secretion pattern and altered cellular metabolism, leading to WAT inflammation and dysregulation of adipogenesis [60,61,68]. Chronic low-grade inflammation of WAT has been extensively reported in individuals with obesity [27,37,41,59,69,70,71,72,73] and associated with adipogenesis inhibition and restriction of the hyperplasic expandability of WAT [37,40,41,53]. In turn, evidence suggests that impaired adipogenesis in scWAT leads to adipocyte hypertrophy [42,62,63], increased proinflammatory cytokine production, and worsened metabolic status [41,42,69,70,72]. While a positive correlation has been reported between adipocyte turnover and insulin sensitivity [53,74], enhanced adipogenesis reduces the number of hypertrophic adipocytes and proinflammatory cytokine production [37]. Studies in rodents have shown that lack of WAT cell plasticity leads to metabolic dysfunction [50,53].

The hypercellularity of WAT is also of great importance for metabolic health. Leptin plays an important role due to its involvement in whole-body energy balance and composition [43,75]. Total fat mass and leptin serum levels are correlated in newborns, children, and adults with and without obesity [75]. Adipocyte hypertrophy has been associated with hyperleptinemia [76]. Therefore, one would expect that serum leptin levels would normalise after weight loss, a state in which smaller adipocyte size has been reported [44,45,46,77]. On the contrary, hypercellularity of WAT, present even after pronounced weight loss, is characterized by a relative leptin deficiency [45,77], which leads to a decrease in energy expenditure and increase in appetite, which have been postulated as some of the many mechanisms involved in weight regain and difficulty maintaining weight loss. 

More recently, it was reported that preadipocyte formation in aSAT and gSAT was correlated with visceral and total abdominal AT and negatively associated with insulin sensitivity [78], challenging the expandability hypothesis. In line with this, others have reported similar findings that challenge the expandability hypothesis [79,80]. Increased adipogenesis and adipocyte turnover may reflect adipocyte fragility and death. A greater cell fragility and death can lead to macrophage recruitment, inflammation, and an unfavourable WAT remodelling [73,78,79], likely contributing to worsened metabolic health. 

Evidence from animal models has shown that the loss of hyperplasic potential in mice leads to metabolic dysregulation. Furthermore, when adipogenesis capacity is restored through either surgical implantation of AT in a lipodystrophy mouse model [81] or pharmacological intervention with thiazolidinediones in an obesity model [82], insulin sensitivity, hyperglycaemia, and hepatic steatosis are improved or even reversed, pointing towards an extremely important role of adipogenesis in metabolic health.

## 5. AT Dynamics during Weight Loss

### 5.1. Lipid Dynamics during Weight Loss

Negative energy balance, in which total energy expenditure is greater than dietary energy consumption, is required for weight loss. An energy deficit can be achieved through hypocaloric dietary plans and exercise and has been shown to be effective in reducing adipocyte size [83,84,85]. Therefore, it would be expected that diet and physical activity have an impact on adipocyte lipid kinetics. Arner et al. [46] tracked WAT lipid kinetics during weight loss in patients with severe obesity undergoing gastric bypass surgery and demonstrated a marked and sustained decrease in BMI over 5 years [46]. The authors reported no differences in mean lipid age when compared at baseline and 5 years after surgery, suggesting that alterations in lipid removal are not central for pronounced weight loss. The authors also reported a significant decrease in adipocyte lipid uptake between years 0 and 5, demonstrating that lipid uptake is the main determinant driving weight loss after bariatric surgery. Interestingly, individuals with the oldest lipids at baseline showed the largest changes in BMI as well as the greatest maintenance of weight lost, whereas those with younger lipid age regained weight within the 5-year follow-up period [46]. Furthermore, the authors reported differences in lipid age between years 0 and 5 among weight-stable and weight-rebound individuals. Lipid age decreased in weight-stable participants and increased in weight rebounders, suggesting that despite not being the main driving mechanism for weight loss, an augmented lipid removal rate contributes to successful maintenance of weight loss in the long-term (Figure 2B). Similar results were obtained in individuals with juvenile or adult-onset obesity [46]. Variations in WAT lipid turnover response to caloric restriction may partially explain the large spectrum of inter-individual variation in weight loss and weight maintenance [46]. Thus, strategies that target adipocyte lipid turnover and lipolysis may be effective in individuals that are resistant to weight loss or maintaining weight loss. Considerable amounts of data show that exercise improves WAT lipolysis [84,86]; therefore, exercise could be a useful strategy for promoting weight loss in individuals with a young lipid age who are more resistant to losing weight and prone to regain it [46].

### 5.2. Adipocyte Turnover during Weight Loss

Spalding et al. studied the effects of marked weight loss on scWAT cellularity in individuals with severe obesity who underwent bariatric surgery [45]. The radical reduction in dietary energy intake as a result of the surgical treatment led to a significant decrease in BMI and scWAT adipocyte cell volume; however, despite the pronounced weight loss, adipocyte number in scWAT was not reduced but remained constant for 2 years after surgery [45]. These findings support the idea that when a higher number of adipocytes is established (such as in childhood obesity), WAT cellular dynamics are adjusted to maintain this number in future life periods, even if significant weight loss takes place (Figure 2B). During adulthood, the total number of adipocytes stays constant; thus, changes in body weight and total boy fat mass mainly occur through changes in adipocyte size [45]. Dietary energy restriction has been effective in reducing weight and adipocyte size [45,83]; nevertheless, WAT cellularity appears to remain constant even after long periods of sustained energy restriction [45]. Therefore, in terms of obesity, current evidence suggests that a preventive approach for the development of this condition, especially during early life, could be of interest.

Exercise is effective in reducing adipocyte TAG content and adipocyte size [84,85]; however, its effects on WAT adipogenesis have been less well characterized. White et al. studied *in vivo* adipogenesis of WAT in sedentary and exercised adult male and female mice [79] and showed that 4 weeks of exercise training led to significantly reduced adipocyte formation in scWAT and vWAT depots [79], suggesting that reduced adipogenesis may be an important exercise-induced mechanism of WAT remodelling. Interestingly, reduced adipogenesis occurred even in the absence of changes in body weight or total AT mass between sedentary and exercised mice, suggesting that exercise itself could also have an impact on WAT cellular dynamics [79]. Importantly, these results were obtained in lean, healthy mice. Therefore, these observations cannot be directly translated to an obesity and/or metabolic dysfunction scenario and further research is needed. 

Differences regarding species should be considered and *in vivo* characterization of WAT remodelling during exercise should be obtained in humans. The long-term effects of exercise in WAT remodelling in obesity and non-obesity conditions, as well as during growth periods, should be assessed to gain a more comprehensive understanding of overall WAT dynamics. Based on these findings, we can speculate that a combination of adequate dietary energy intake and adequate physical activity could have a positive impact not only in preventing the development of obesity, but also in treating this condition by reducing adipocyte lipid uptake, improving lipolysis, and possibly promoting a lower adipogenesis rate. 

## 6. Conclusions

WAT exhibits remarkable plasticity, adjusting its size in response to environmental stimuli. WAT mass and size are regulated by cellular and lipid turnover. Current evidence suggests that childhood and puberty play pivotal roles in the proper development of WAT. The development of WAT during these critical life stages is profoundly influenced by environmental factors, particularly energy intake.

In adulthood, unlike in childhood, hyperplasia plays a minimal role in expanding fat mass. The turnover of adipocytes remains relatively constant during adulthood, and neither weight gain nor weight loss appear to significantly affect the number or cellular kinetics of adipocytes in WAT. During this phase, alterations in fat mass primarily arise from variations in the rate at which lipids are removed from adipocytes. However, the rate of lipid removal from WAT diminishes during aging, leading to weight gain through the accumulation of fat mass. This process also involves the uptake and storage of lipids within individual adipocytes, leading to their enlargement and contributing to overall fat accumulation. 

During weight loss, AT primarily reduces fat cell size while maintaining adipocyte number, even when reaching a non-obese state. Decreased adipocyte lipid uptake is the main mechanism underlying fat loss. Therefore, nutrient intake and energy play a crucial role in adipocyte lipid uptake and overall WAT lipid dynamics.

In conclusion, studies on AT dynamics and lipid turnover in health and obesity provide valuable insight into the complex mechanisms underlying AT function. One of the limitations of our understanding of AT lipid turnover in humans is the slow rate of turnover and methodological constraints. However, understanding the intricate interplay between AT dynamics and lipid metabolism is crucial for developing targeted interventions and therapeutic strategies to combat obesity and its associated health risks. Further studies combining detailed clinical phenotyping with measures of regional adipose cell and lipid turnover are needed in humans to firmly establish their role in the pathophysiology of metabolic disorders. Continued research in this field holds great promise for advancing our understanding of AT biology and ultimately improving the management and treatment of obesity-related disorders.

## Data Availability

No new data were created or analyzed in this study. Data sharing is not applicable to this article.

## Abbreviations

AT: adipose tissue; APC, adipocyte progenitor cell; BMI, body mass index; BAT, brown adipose tissue, cAMP, cyclic adenosine monophosphate; CVD, cardiovascular disease, DNL, de novo lipogenesis; FCHL, familial combined hyperlipidaemia; HFD, high-fat diet; LDL, low-density lipoprotein; mRNA, messenger ribonucleic acid; TAG, triacyclglycerol; scWAT, subcutaneous tissue; aSAT, abdominal subcutaneous adipose tissue; gSAT, gluteofemoral subcutaneous adipose tissue; SVF, stromal vascular fraction; UCP1, uncoupling protein 1; VLDL, very-low-density lipoprotein; vWAT, visceral white adipose tissue; WAT, white adipose tissue.
